# The Impact of HIV- and ART-Induced Mitochondrial Dysfunction in Cellular Senescence and Aging

**DOI:** 10.3390/cells10010174

**Published:** 2021-01-16

**Authors:** Madison Schank, Juan Zhao, Jonathan P. Moorman, Zhi Q. Yao

**Affiliations:** 1 Center of Excellence in Inflammation, Infectious Disease and Immunity, James H. Quillen College of Medicine, East Tennessee State University, Johnson City, TN 37614, USA; niecem@etsu.edu (M.S.); zhaoj2@etsu.edu (J.Z.); moorman@etsu.edu (J.P.M.); 2 Division of Infectious, Inflammatory and Immunologic Diseases, Department of Internal Medicine, Quillen College of Medicine, East Tennessee State University, Johnson City, TN 37614, USA; 3 Hepatitis (HCV/HBV/HIV) Program, James H. Quillen VA Medical Center, Department of Veterans Affairs, Johnson City, TN 37614, USA

**Keywords:** HIV, ART, mitochondria, mtDNA, ROS, cellular dysfunction

## Abstract

According to the WHO, 38 million individuals were living with human immunodeficiency virus (HIV), 25.4 million of which were using antiretroviral therapy (ART) at the end of 2019. Despite ART-mediated suppression of viral replication, ART is not a cure and is associated with viral persistence, residual inflammation, and metabolic disturbances. Indeed, due to the presence of viral reservoirs, lifelong ART therapy is required to control viremia and prevent disease progression into acquired immune deficiency syndrome (AIDS). Successful ART treatment allows people living with HIV (PLHIV) to achieve a similar life expectancy to uninfected individuals. However, recent studies have illustrated the presence of increased comorbidities, such as accelerated, premature immune aging, in ART-controlled PLHIV compared to uninfected individuals. Studies suggest that both HIV-infection and ART-treatment lead to mitochondrial dysfunction, ultimately resulting in cellular exhaustion, senescence, and apoptosis. Since mitochondria are essential cellular organelles for energy homeostasis and cellular metabolism, their compromise leads to decreased oxidative phosphorylation (OXPHOS), ATP synthesis, gluconeogenesis, and beta-oxidation, abnormal cell homeostasis, increased oxidative stress, depolarization of the mitochondrial membrane potential, and upregulation of mitochondrial DNA mutations and cellular apoptosis. The progressive mitochondrial damage induced by HIV-infection and ART-treatment likely contributes to accelerated aging, senescence, and cellular dysfunction in PLHIV. This review discusses the connections between mitochondrial compromise and cellular dysfunction associated with HIV- and ART-induced toxicities, providing new insights into how HIV and current ART directly impact mitochondrial functions and contribute to cellular senescence and aging in PLHIV. Identifying this nexus and potential mechanisms may be beneficial in developing improved therapeutics for treating PLHIV.

## 1. Introduction

### 1.1. Human Immunodeficiency Virus (HIV)

**Figure 1:**
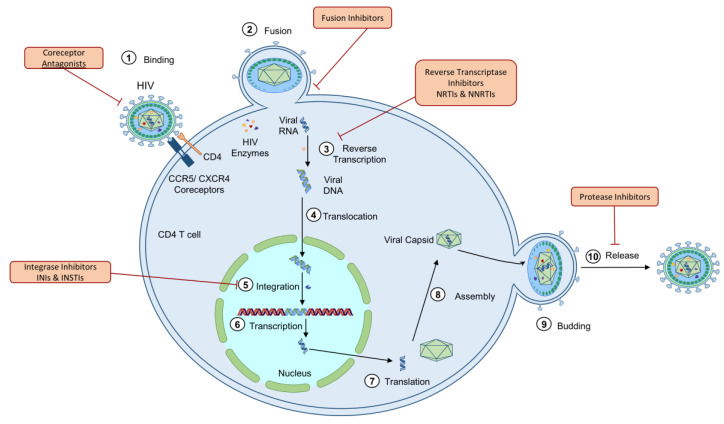
The viral lifecycle of human immunodeficiency virus HIV-1 infection and steps of antiretroviral therapy (ART) interference. HIV infection begins by glycoproteins, such as Env/gp120, on the surface of the HIV virus interacting with CD4 and CCR5/CXCR4 receptors on the surface of a target CD4 T cell (step 1), which is inhibited by coreceptor antagonists. This facilitates fusion between the HIV membrane and the cell membrane (step 2). This step is inhibited by fusion inhibitors. The viral capsids containing HIV enzymes and viral RNA are released into the cell cytoplasm, followed by reverse transcription of the viral RNA into DNA by HIV reverse transcriptase (step 3), which is inhibited by nucleoside-analog and non-nucleoside reverse transcriptase inhibitors (NRTIs and NNRTIs). The viral DNA is then transported into the cell nucleus for integration into the host genome by HIV integrase and transcription (steps 5 and 6). Integrase inhibitors, including INIs and INSTIs, prevent the integration step. The transcribed DNA is then released into the cytoplasm for translation via Rev-mediated export (step 7) and assembly into the viral capsid (step 8) for budding (step 9) and release (step 10), following which HIV protease cleaves polyproteins to create an active virus capable of infecting new cells. Protease inhibitors prevent the final release of a functional replicating virus.

HIV is a lentivirus of the Retroviridae family, infecting humans worldwide. The HIV lifecycle has seven major steps: binding, fusion, reverse transcription, integration, replication, assembly, and budding (Figure 1) [1,2,3,4,5,6,7,8]. The mechanism of HIV infection is best described as an opportunistic, strategic manipulation of host cells, inducing immense deregulation on the human immune system, including intrinsic, innate, and adaptive immunity, as well as on many nonimmune cells and tissues [6]. HIV infection is characterized by a progressive weakening of the immune system, induced by a significant depletion of CD4 T cells. Two main factors contribute to the loss of CD4 T lymphocytes: (1) the aberrant inflammatory response induced by HIV infection; and (2) viral pathogenesis—HIV uses cells for viral integration, replication, and release [7,8,9]. HIV infection also induces a significant upregulation of proinflammatory cytokines, further contributing to the viral pathogenesis [10,11,12,13].

One of the major difficulties in curing HIV is reservoir formation and viral latency. Latency is a reversible quiescent state during which the virus survives in host cells but does not replicate, concealing itself in reservoirs through which it can later become reactivated [14]. HIV infects target cells, including but not limited to CD4 T cells, macrophages, and lymphoid tissues, and integrates proviral DNA into the host genome to establish reservoirs. Antiretroviral therapy (ART) allows for virological control of HIV replication and prevents CD4 T cell numbers from falling below the threshold (200 cells/μL) associated with severe immune deficiency; however, ART cessation readily allows for activation of reservoir-residing cells and the production of actively replicating HIV [11,15,16,17,18].

The current leading form of treatment for HIV is a form of combined ART therapy, termed highly active antiretroviral therapy (HAART) [19,20]. During the initial development of ART, treatments were used in a monotherapy form; however, the increased incidence of drug resistance led to the use of combination therapy to minimize morbidity and mortality of HIV-infection and efficiently control viral replication [21]. ART is highly successful in reducing actively replicating virus; however, due to the presence of HIV reservoirs and incomplete elimination of virus, once ART is stopped, viremia rises to levels similar to those observed prior to ART treatment [22]. No currently available treatment successfully clears integrated proviral DNA incorporated in the host genome [15,23,24,25]. Thus, PLHIV must maintain strict lifelong treatment adherence in order to prevent viral rebound [26]. Additionally, despite ART-mediated viral suppression aiding CD4 T cell recovery, this reversal constitutes an incomplete immune reconstitution, forcing PLHIV to face lifelong challenges with immune failure [27,28].

### 1.2. Mitochondria Function in Cellular Energetics and Homeostasis

Mitochondria are organelles present in almost all eukaryotic cells and provide energy to the cell in order to maintain normal cellular functions and homeostasis [29]. Mitochondria complete oxidative phosphorylation (OXPHOS) to form ATP via mitochondrial respiration through electron transport chain (ETC)-mediated production of an electrochemical gradient across the inner mitochondrial membrane, termed the mitochondrial membrane potential (ΔΨ_m_). The presence of this gradient enables ATP synthase to recycle the protons from the gradient to facilitate the energetically unfavorable production of ATP [30,31,32,33]. Any slight disruption to the ΔΨ_m_ has large consequences for the levels of cellular respiration and ATP synthesis.

Mitochondria are also involved in apoptosis signaling pathways and calcium signaling and storage. Mitochondria are integral to both intrinsic and extrinsic cell death pathways through several signaling molecules, including cytochrome *c* and caspases [34]. Additionally, mitochondria are essential in the regulation of cellular metabolism, immune signaling, cell differentiation, and maintenance of the cell cycle and cell growth, all of which contribute to mitochondrial biogenesis and the fine balance between mitochondrial fusion, fission, and morphology [32,35,36]. Mitochondrial morphology and function have been shown to become disrupted in several disease states. Deregulation of mitochondrial function leads to increased reactive oxygen species (ROS), which are involved in cell signaling but also induce toxicity and DNA damage [29,35]. Increased ROS production due to deregulated ETC activity leads to the upregulation of uncoupling proteins and protein leakage through the adenine nucleotide translocator. These phenomena lead to the uncoupling of mitochondrial-mediated respiration and ATP synthesis, increasing mitochondrial oxygen consumption and fatty acid oxidation. Despite increased oxygen consumption, ATP synthesis is limited, resulting in ATP deficiency and deregulated cellular metabolism [36,37].

Mitochondria have an independent circular DNA composed of ~16 kilobases. While the majority of the genes encoding for proteins required for mitochondrial function are encoded in the nuclear genome and their proteins are transported into the mitochondria, mitochondrially encoded genes are essential for mitochondrial functions. Specifically, mitochondrial DNA (mtDNA) encodes for crucial components of the ETC and mitochondrial biogenesis. Mutations and deletions of mtDNA, which can be caused by ROS, lead to adverse effects on mitochondrial function [38,39,40]. This process may contribute to aging and the gradual loss of vigor in aged populations [41,42]. For example, mitochondrial deregulation leads to leakage of high-energy electrons in the respiratory chain, creating ROS. Increased oxidative stress leads to drastic increases in DNA mutations or deletions, creating a cycle of mitochondrial deregulation, increased oxidative stress, and mtDNA damage. Nuclear DNA repair pathways, such as base excision repair and double-strand break repair, can repair damaged mtDNA through nuclear-encoded genes; however, these processes may be deregulated during HIV-infection. For instance, emerging work from our group and others have shown dysregulation of critical DNA damage response proteins, such as ATM, ATR, DNA-PKcs, TOP I/IIα, and p53 in the setting of HIV-infection [39,43,44,45,46,47,48]. The literature shows that mitochondrial function and signaling pathways become disrupted in the presence of HIV-infection and ART treatment.

### 1.3. ART-Suppressed HIV and Mitochondrial Dysfunction in Cellular Senescence and Aging

Recently, several works have classified multiple physiological characteristics as hallmarks of aging, including genomic instability, telomere attrition, mitochondrial dysfunction, reduced proliferative capacity, altered intracellular communication, aberrant inflammation, and cellular senescence (the quiescent, nonreplicative state in which cells are functionally anergic), all of which are commonly observed in PLHIV [49,50,51]. Telomere erosion, along with telomeric DNA damage, has been deemed a molecular clock of cellular aging. Telomeric DNA damage activates p53, leading to cellular growth arrest, apoptosis, and senescence [52]. In addition, a study examining ART-treated, aviremic PLHIV identified a correlation between shortened telomere length and poor immunologic recovery [53]. Interestingly, recent studies suggest that senescent cells secrete mediators that can induce mitochondrial dysfunction in bystander neighboring cells, leading to increased ROS production and DNA damage to induce senescence in the form of senescence-induced senescence rather than replicative senescence [54,55,56]. Senescence has also lately been identified as a potential antiviral defense mechanism to aid in activating the immune system to clear the infection. However, prolonged exposure to cellular damage that occurs during HIV infection leads to an increase in cellular senescence, which ultimately leads to an accumulation of chronic inflammation and immune failure [57,58]. Each of these factors ultimately contributes to increased incidence of age-related comorbidities, such as neurodegenerative, cardiovascular, and metabolic diseases, and cancers, in PLHIV [51,53,59,60,61].

In addition to HIV-accelerated premature aging, ART-treatment may also contribute to mitochondria-related aging. While early ART initiation and strict ART adherence have been shown to reduce the risk of PLHIV developing comorbidities, immune phenotyping has also revealed that even in cases of long-term ART-treated individuals still demonstrate abnormal cellular functions, homeostasis, and cell activation status, implying the possibility that ART may further contribute to the HIV-accelerated premature aging [51,61]. Specifically, ART has been shown to induce low-grade inflammation, mitochondrial dysfunction, and senescence in various cell types, even after relatively short-time exposure [62]. These data highlight the need for further investigation into HIV- and ART-induced mitochondrial compromise and inflammaging (inflammation-induced aging).

## 2. Current Outlook

Considering the number of essential processes mitochondria are involved in, any influence from external stimuli, such as HIV infection or ART treatment, may have a detrimental effect on mitochondrial activity. The deregulation of mitochondrial function has several downstream effects, many of which are critical to cellular function and survival. The current leading measures of mitochondrial functions include evaluation of mtDNA copy number, mtDNA mutations, ROS generation, ΔΨ_m_, cellular respiration, apoptosis, and ATP production. These parameters can be used to assess both the metabolic and genomic status of cells following potential drug and HIV toxicity. There is a current lack of understanding of how ART treatments and HIV individually and potentially cooperatively lead to mitochondrial dysfunction, as well as how these processes likely contribute to the progressive immune aging observed in PLHIV on ART.

### 2.1. HIV-Induced Mitochondrial Dysfunction: The Influence of Virally Encoded Proteins

HIV infection has been shown to induce differential regulation of mitochondrial activity in various cell types. For instance, studies have shown that ΔΨ_m_ is reduced in HIV-infected, ART-naïve patients compared to HIV-negative healthy subjects, and a negative correlation between ΔΨ_m_ and the percentage of apoptotic lymphocytes. CD4^+^ T cell numbers have been shown to be positively correlated to the change in ΔΨ_m_ in HIV-infected, ART-naïve individuals [63,64]. It is likely that mitochondrial deregulation partially arises from the activities of virally encoded proteins. HIV-1 encodes several proteins critical for viral replication and integration, including viral structural proteins Gag, Pol, and Env, essential regulatory elements Tat and Rev, and accessory regulatory proteins Nef, Vpr, Vif, and Vpu. Literature shows that several of these virally encoded proteins, including Env, Vpr, Tat, Nef, and Vpu, contribute to HIV-induced cell apoptosis, which is mediated by mitochondrial activity [65,66,67]. Each of these proteins can cause bystander-induced apoptosis, during which infected cells expressing virally encoded proteins can interact with neighboring uninfected cells to induce apoptosis. This process is one of the most widely accepted hypotheses regarding how HIV-infection causes depletion of CD4^+^ T cells at a rate inconsistent with viremia levels [11,63,65,66,67]. Thus, deregulation of the ΔΨ_m_ and cellular apoptosis by HIV greatly influences mitochondrial homeostasis.

### 2.2. HIV-Encoded Env: A Regulator of Viral Infection, Apoptosis, and Mitochondria

**Figure 2:**
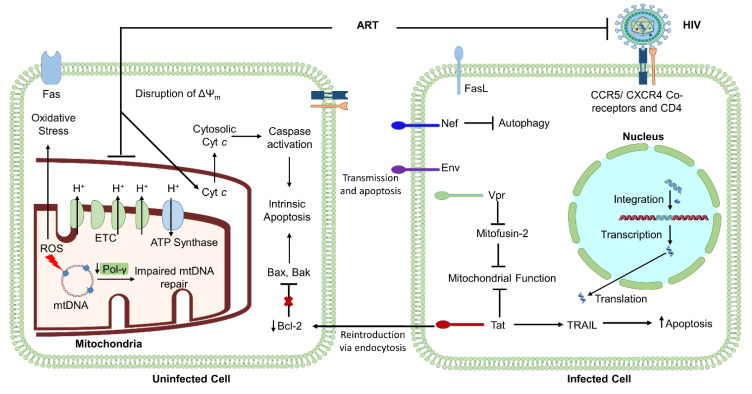
Model of cooperative induction of mitochondrial compromise by HIV-infection and ART-treatment. Mitochondrial dysfunction is mediated by viral integration, disruption of mitochondrial membrane potential (ΔΨm), decreased respiration and ATP synthesis, mtDNA mutations, impaired mtDNA repair, intrinsic apoptosis, and oxidative stress. Collectively, these deregulations facilitate energetic failure and depletion of CD4 T cells.

The HIV-gene *env* encodes for the viral envelope forming protein Env, which has been widely shown to be involved in influencing cell apoptosis. Specifically, the *env* gene codes for a 160 kD glycoprotein, which is cleaved into noncovalently linked gp41 and gp120. The N-terminal subunit, gp120, exists outside of the viral membrane, facilitating transient interaction with coreceptors such as CXCR4 and CCR5 on target cells. This interaction mediates entry into cells to establish infection, but also often leads to cell death through mitochondrial-facilitated apoptosis, mtDNA instability, and irregular calcium transport and signaling [68,69]. Gp120 elicits apoptosis via depolarization of the mitochondrial membrane, which leads to disrupted ETC activity, ATP synthesis, and gluconeogenesis, ultimately resulting in the release of cytochrome *c,* activating caspases 9 and 3 to result in the activation of cellular apoptosis (Figure 2).

In addition to facilitating infection via interactions with coreceptors and the glycoproteins present on the virion surface, Env is also critical for interaction with uninfected neighboring cells via its localization in the cell membrane of an infected cell and interaction with neighboring cell surface receptors (see Figure 2) [70,71]. Thus, the connection between bystander-induced apoptosis and cells presenting virally encoded proteins may provide key insights into the mechanism by which HIV-infection leads to diminished numbers of CD4^+^ T cells and premature immune aging [71,72,73].

### 2.3. HIV-Encoded Vpr: A Regulator of Apoptosis and Mitochondrial Function

In addition to mitochondrial deregulation induced by virally-encoded Env, Vpr is also capable of eliciting HIV-induced apoptosis. Vpr is an accessory protein that is essential to the progression of viral integration via facilitating transportation of the preintegration complex into the host cell nucleus to allow for the incorporation of the viral genome into host DNA [74,75]. The ability of the Vpr gene product to induce cellular apoptosis arises from Vpr mediated activation of the intrinsic death pathway via cytochrome *c* and caspase activation, similar to Env activity [72,76]. Furthermore, a systematic mutation of the open reading frames (ORFs) of HIV-1 revealed that Vpr, in conjunction with Vif, was able to induce cell death and G2 cell cycle arrest [73].

Studies have also demonstrated Vpr-induced mitochondrial compromise via damage to mitochondrial fusion protein mitofusin 2 (Mfn2). Mfn2 critically functions to regulate mitochondrial functions such as contact with other cellular organelles, trafficking, turnover, and fusion. Vpr-induced damage to Mfn2 thus alters Mfn2-mediated interactions between the endoplasmic reticulum (ER) and mitochondria in human peripheral blood mononuclear cells (PBMCs) (Figure 2). Vpr can localize to the ER, mitochondria-associated membranes (MAM), and mitochondrial outer membrane (MOM), likely occurring through the integration of the C-terminal transmembrane domain. In addition to disrupting the structural stability of the MOM, Vpr was also shown to correlate with reduced protein expression levels of Mfn2 [77]. This level of Vpr-induced repression of Mfn2 corresponds to significant depletion of mitochondrial activity and is likely involved in the pathogenesis of a wide range of disease states.

### 2.4. HIV-Encoded Tat: A Regulator of Apoptosis and DNA Damage Repair

The HIV genome also encodes for the Tat protein, which is known to be produced in infected cells, followed by release and reuptake into uninfected neighbor cells via endocytosis (Figure 2). Tat is also capable of triggering apoptosis and taking part in the bystander effect and infection of neighboring cells through CCR5 and CXCR4 receptors. Literature suggests that Tat induces apoptosis via regulation of the classical intrinsic apoptosis pathway. Specifically, Tat can downregulate Bcl-2, an antiapoptotic protein, and upregulate the proapoptotic caspase-8 to facilitate the release of proapoptotic agent cytochrome *c*, as well as proapoptotic FasL and Bax [78]. Literature also shows that Tat upregulates TNF-related apoptosis-inducing ligand (TRAIL) production in macrophages (Figure 2) and that T cells from PLHIV are more prone to TRAIL-induced apoptosis [79].

Furthermore, Tat has been shown to mechanistically inhibit the telomerase enzyme. Telomerase repairs DNA shortening that occurs with continual replication by extending the hexameric DNA repeats at the ends of chromosomes—telomeres. While telomerase has been shown to be reduced in CD4 T cells infected with HIV, Tat exposure alone was able to induce this telomerase reduction. Specifically, Tat can reduce nuclear expression of the catalytic subunit of telomerase, telomerase reverse transcriptase (hTERT), and disturb the AKT pathway, which is critical for activation of hTERT [80]. Tat also has been shown to suppress the transcription of cell cycle regulator p53 [81,82]. These data highlight an additional mechanism by which Tat is able to damage the replicative potential of both infected and noninfected CD4 T cells, potentially contributing to the depletion of functional CD4 T cells in PLHIV. Additionally, some studies have identified a potential molecular link between telomeric DNA damage and mitochondrial dysfunction via a p53-dependent pathway [54,83,84]. Collectively, these findings indicate that Tat facilitates mitochondrial dysfunction via several independent mechanisms.

### 2.5. HIV-Encoded Nef: A Regulator of Apoptosis and Mitophagy

The virally encoded protein Nef is essential for T cell activation and maintaining persistent infection [85]. Nef induces TCR-independent activation of T cells and allows for the infection of neighboring T cells through the CCR5 and CXCR4 receptors, contributing to bystander-induced apoptosis and depletion of CD4^+^ T cells. Literature shows that T cells expressing Nef also express FasL, enabling these cells to kill uninfected T cells expressing Fas, indicating that Nef has an additional method to induce bystander apoptosis. Nef also induces apoptosis via two proapoptotic structural motifs [79].

Moreover, Nef has been shown to regulate the processes of autophagy and mitophagy (the selective degradation of damaged mitochondria via autophagy). Most commonly, Nef inhibits autophagy to allow for viral survival by interacting with integral proteins in autophagy initiation, including Beclin 1 [65,86]. Since autophagy is critical for maintaining cellular homeostasis and mitochondria are the key energetic supplier, continued inhibition of autophagy and mitophagy from Nef interactions may lead to disruption of mitochondrial activity and accumulation of nonfunctional mitochondria [87]. This would further lead to increased ROS and mitochondrial DNA damage.

### 2.6. HIV-Mediated Mitochondrial Compromise

Individuals with latent HIV show enrichment of genes regulating cell death, cell activation, and inflammatory chemokines and cytokines in PBMCs and muscle and adipose tissues, whereas genes responsible for governing mitochondrial function and biogenesis were downregulated in both PBMCs and adipose tissues from patients with long-term controlled HIV viral load [65,88]. Thus infection also has a direct influence on mitochondrial function, activation of immune cells, and inflammation, all of which likely contribute to the premature aging observed in HIV infection [64,83]. It is important to note that HIV-induced apoptosis via depolarization of the mitochondrial membrane also contributes to mitochondrial dysfunction by inducing mtDNA mutations and deregulating OXPHOS, ATP synthesis, and ROS production [64]. The ΔΨ_m_ is essential to maintaining cell viability and homeostasis, as evidenced by ΔΨ_m_ disruption, leading to failure to eliminate dysfunctional mitochondria and transport critical ions and proteins required for mitochondrial activity [33,88].

Additionally, research shows that PLHIV are more likely to show premature aging, which is linked to increased oxidative stress and damage and shortened telomeres which could further contribute to mitochondrial and metabolic compromise [52,84,89,90,91,92]. A study investigating mitochondrial function in ART-naïve and ART-exposed PLHIV demonstrated increased mitochondrial mass in both CD4^+^ and CD8^+^ T cells with the loss of CD4^+^ T cells. HIV also appeared to target ΔΨ_m_ in CD8^+^ T cells, leading to ROS accumulation in CD4^+^ T cells. Following ART therapy, both cell subsets showed a significant decrease in mitochondrial mass, followed by an increase after three years of treatment. These results indicate a dynamic fluctuation in immune cell response to HIV infection and ART-mediated suppression of infection, which was unique between CD4^+^ and CD8^+^ T cells [7]. This deregulation of mitochondrial parameters and increased oxidative stress can initiate a vicious cycle of overproduction of ROS, mtDNA damage, mitochondrial failure, and apoptosis, further contributing to metabolic compromise during HIV infection.

Interestingly, a study examining the intracellular distribution of HIV-1 RNA in infected cells revealed significantly increased levels of viral RNA localized in the mitochondria compared to the cytoplasm and nucleus in the latently HIV-1-infected T cell line ACH2 following TCR stimulation. The level of viral RNA localized in mitochondria of infected cells was significantly lower in chronically infected cells versus acutely infected cells [93]. Live-cell real-time fluorescence imaging also showed that mitochondria from HIV-infected H9 cells can be transported into uninfected cells (MT2 target cells) to facilitate viral transmission. Furthermore, culturing uninfected cells in the presence of isolated mitochondria from HIV-infected T cells resulted in infection, as measured by viral antigen p24 production, syncytia development, and depletion of target cells, which was prevented by culturing in the presence of pharmacological inhibitors of mitochondrial function [94]. Mitochondria from HIV-infected cells can thus function as viral reservoirs to facilitate cell-to-cell infection. Additionally, a study identified that PLHIV who have self-control of their infection and low levels of viremia, referred to as long-term nonprogressors (LTNPs), have significantly lower rates of apoptosis and mitochondrial compromise in PBMCs compared to untreated asymptomatic typical progressors (TPs) with high viremia and immunological deterioration [95]. These results indicate the existence of a potential conditional influence of mitochondria in HIV infection, viral latency, and disease severity. Thus, it may be possible that HIV intentionally hijacks mitochondrial activity to facilitate increased viral transmission and infectivity at varying levels during different stages of HIV-infection.

The modulation of mitochondrial function may be an ideal approach for the virus to facilitate infection due to the critical role of mitochondria in innate and antiviral immune responses [96]. To avoid mitochondria-mediated HIV-clearance, HIV likely targets mitochondrial function via mtDNA copy number depletion and deregulation of ΔΨ_m_ and mitochondrial signaling. Collectively, the influences from HIV-infection contribute to both viral infectivity and ultimate mitochondrial compromise. This indicates the presence of a fine-tuned balance between using mitochondrial for transmission and for inhibiting immune responses to HIV infection.

### 2.7. ART-Induced Mitochondrial Dysfunction

While mitochondrial functions are repressed in ART-naïve PLHIV, prolonged exposure to ART has been shown to increase the adverse effects previously thought to only be associated with HIV infection [21,83,97,98,99,100,101,102,103,104,105]. In fact, in the era of ART, the impact of progressive infection is lesser than that of long-lasting ART treatment, especially in the case of aviremic patients. Classes of ART approved for the treatment of HIV include nucleoside-analog reverse transcriptase inhibitors (NRTIs), non-nucleoside reverse transcriptase inhibitors (NNRTIs), protease inhibitors (PIs), integrase inhibitors (INIs), which contains the subclass integrase strand transfer inhibitors (INSTIs), fusion inhibitors, and coreceptor antagonists, each of which interferes with critical steps in the viral replication lifecycle. The first generation of approved ART drugs was NRTIs. NRTIs inhibit DNA polymerase gamma (Pol-γ), which functions in mitochondrial DNA (mtDNA) replication and maintenance, implicating ART as a potential cause of mitochondrial dysfunction [102]. Alternately, mitochondrial dysfunction is also associated with NNRTIs, PIs, and INSTIs, despite these drug classes not disrupting Pol-γ activity [71,95,106]. Despite ART treatment decreasing HIV-associated comorbidities and improving mortality of PLHIV, mitochondrial functions are disrupted even in clinically stable patients [103].

The administration of combined ART creates an increasingly difficult landscape to assess the disruption of mitochondrial function by ART regimens individually. Understanding the unique processes by which HIV-infection in combination with ART leads to mitochondrial dysfunction may allow for the development of improved therapeutics and quality of life for PLHIV. Considering the prevalence of the inclusion of NRTIs, NNRTIs, PIs, and INSTIs, as well as the evidence supporting increased adverse effects of these drugs compared to fusion inhibitors and coreceptor agonists, for the purposes of this review we hereafter focus on mitochondrial toxicity associated with NRTIs, NNRTIs, PIs, and INSTIs [107,108]. These results, along with mitochondrial deregulation by all ART classes, are summarized in Table 1.

### 2.8. NRTIs

Currently, NRTIs are the most common drug class to be incorporated in ART. NRTIs are incorporated into viral DNA and cause chain termination to disrupt the viral capacity to complete reverse transcription of viral RNA into DNA (step 3 in Figure 1) [21,95,101,106]. NRTIs may be incorporated into mtDNA via Pol-γ by competing with natural thymidine triphosphates, ultimately leading to mutations in the chain or termination of mtDNA synthesis [122,123,124,125,126]. However, more recent studies have highlighted additional mechanisms in NRTI-induced mitochondrial dysfunction [127]. NRTIs have been shown to impair ATP/ADP translocation through channels, which inhibits the coupling of respiration and ATP synthesis [30,114]. NRTIs also induce time- and dose-dependent depletion of mtDNA copy number, lymphocyte proliferation, and cytochrome c-oxidase II expression in primary CD4^+^ and CD8^+^ T lymphocytes from HIV-negative individuals [113]. NRTI treatment has also been shown to lead to a significant upregulation of coding region nonsynonymous substitutions in mtDNA in PLHIV, leading to mitochondrial toxicity, compared to HIV-negative controls. Results revealed significantly increased mtDNA deletion mutations in the *ND4* gene, which codes for NADH dehydrogenase, in treated PLHIV compared to uninfected healthy subjects [106]. NRTI-induced mitochondrial dysfunction was also observed to be progressive and continued NRTI-exposure was associated with progressive loss of oxidative respiration in primary human lymphocytes [113]. Furthermore, mitochondrial function and mtDNA content, along with associated pathologies such as neuropathy and hyperlactatemia have been shown to recover upon NRTI discontinuation. Thus, the current recommendation for individuals experiencing mitochondrial toxicity following NRTI treatment is the discontinuation of the treatment [100,128,129,130]. These results indicate that the reduction in mtDNA copy number is significantly linked to the decline in mitochondrial respiration capabilities. This phenomenon is likely due to mtDNA copy number reduction initiating ineffective synthesis of OXPHOS proteins, increasing the level of oxidative stress produced by mitochondria that induce further damage to mtDNA, proteins, and lipids [127]. 

NRTIs, but NNRTIs, have also been shown to inhibit telomerase function both in vitro and in vivo, while also being incorporated into telomeric DNA, leading to chain termination and often cell death [131]. Furthermore, Pol-γ has also been shown to be sensitive to oxidative stress [111]. Together, these data represent a molecular cycle in which mtDNA mutations, which may be increased due to the high processivity of Pol-γ and ART exposure, lead to energetic deprivation and mitochondrial dysfunction, inducing mitochondrial function failure and increased oxidative stress. ROS are then able to induce further mtDNA damage and impair mtDNA replication. By this mechanism, the level of mtDNA mutations will continue increasing and may lead to a progressive system of increasing symptoms. Interestingly, a study identified that HAART-treated PLHIV had significantly higher serum oxidant levels and reduced serum antioxidant makers compared to both untreated PLHIV and uninfected controls. Furthermore, this level of increased oxidative stress was directly correlated with the level of HAART treatment adherence [132]. While this does not directly implicate NRTIs alone (all individuals were on various forms of combined HAART, but all included at least one NRTI), it highlights a critical role HIV-treatments in inducing oxidative stress in ART-treated individuals. Collectively, these data suggest that mitochondrial toxicity induced by NRTI treatment may arise due to the deregulation of the mitochondrial network, mtDNA mutations, and oxidative stress.

### 2.9. NNRTIs

NNRTIs inhibit reverse transcription of the virus via binding to a hydrophobic pocket adjacent to the active site of HIV reverse transcriptase (step 3 in Figure 1) [21,95,101,106]. The most common NNRTI is efavirenz (EFV), which is particularly successful in inhibiting viral replication. However, EFV is correlated with reduced cell growth and increased apoptosis, oxidative stress, mitochondrial mass, and mitochondrial protein expression in Hep3B hepatic cells. Several of these results were obtained even after short incubation times with EFV. This indicates the likelihood that these modifications are not directly linked to mtDNA replication, due to the short incubation times and lack of change in mtDNA copy number. Additionally, the disruption to mitochondrial function was rescued by treatment with antioxidant Trolox, highlighting the critical role of ROS in generating mitochondrial compromise [109]. EFV is also toxic to hepatocytes and human coronary artery endothelial cells and disrupts the ΔΨ_m_, leading to cytochrome *c* release and apoptosis [116,117]. Both CD4^+^ and CD8^+^ T cells show decreased levels of activation markers after NNRTI treatment, indicating a weakening of the immune system and ability to mount an immune response after NNRTI treatment [133].

A study in Jurkat cells evaluating the effects of exposure to the NRTI zidovudine (AZT) or the NNRTI EFV revealed that EFV, but not AZT, significantly upregulated apoptosis, the release of cytochrome *c*, and reduced ΔΨ_m_ in a dose- and time-dependent manner [110]. Importantly, while the evaluation of mitochondrial alterations induced by other NNRTIs, such as nevirapine (NVP), does not align linearly with the results obtained for EFV, NVP has been shown to induce apoptosis via deregulation of ΔΨ_m_ in lymphocytes from PLHIV [115]. Collectively, these data indicate that while different NNRTIs may not induce the same types of mitochondrial dysfunction based solely on their mechanism of action, these drugs can still induce differential regulation of various mitochondrial functions and cellular stress.

### 2.10. PIs

In addition to NRTIs and NNRTIs, another common drug class included in HAART are PIs. PIs interfere with the cleavage of essential viral maturation polyprotein precursors, including gag and pol by inhibiting the HIV protease (step 10 in Figure 1) [21,95,101,106]. There is a significant amount of data supporting the role of PIs in inducing oxidative stress among a large number of cell types and model systems, ultimately leading to the main side effects associated with PIs, including cardiovascular disease and lipodystrophy, which have been reviewed elsewhere [118,122]. Monotherapy of CD4 T cells with the PI ritonavir led to increased mtDNA copy numbers in CD4^+^ T cells, increased ROS, and an interruption to ΔΨ_m_ and thus ETC and OXPHOS capacity [64]. Furthermore, ritonavir treatment decreased ΔΨ_m_, increased NADPH oxidase subunits, increased oxidative stress, decreased ATP production, and deregulated MAPK ERK1/2 activity in macrophage-derived foam cells [119]. Ritonavir treatment of Huh-7.5 (human hepatoma cells) showed increased ER stress even after only 6 h of exposure. This ER stress also triggered the translocation of BAX to mitochondria to initiate apoptosis independently of mitochondria (there was no change in mitochondrial potential or release of cytochrome *c*). Additionally, it was confirmed that both EFV and ritonavir were able to enter the mitochondria and lead to a reduction of the ΔΨ_m_, leading to BAX translocation and cytochrome *c* and ultimately apoptosis [119,122,123]. PIs are also tightly correlated with metabolic disruption of lipid metabolism and glucose transport, which are also controlled by mitochondria [134,135]. Another PI implicated in mitochondrial dysfunction is atazanavir, which was shown to induce autophagy, apoptosis, mitochondrial superoxide generation, and depolarization of the membrane potential, while ritonavir caused apoptosis but low rates of autophagy in preadipocytes [136].

PIs have also been identified to induce cell death in PBMCs from uninfected individuals. While some studies have shown decreased apoptosis in HIV-infected and uninfected T cells following treatment with PIs [137,138], another study evaluating T-cell proliferation and cell death following exposure of T cells from healthy individuals to PIs indinavir (IDV) and saquinavir (SQV) revealed decreased proliferation, reduced ΔΨ_m_, and increased CD4 T cell death [139]. Conversely, PIs have also been shown to inhibit apoptotic pathways in HIV infected T cells, specifically by inhibiting apoptosis induced by gp120; however, PIs also contribute to a disrupted mitochondrial membrane and induce necrosis [102,140]. These data indicate that PIs may demonstrate both advantageous and detrimental effects in a cell- and tissue-dependent manner.

### 2.11. INSTIs

INSTIs have become a widely used ART formulation. INSTIs inhibit DNA strand transfer, during which the viral DNA ends are joined into host DNA (step 5 in Figure 1) [21,95,101,106]. In recent years, the most common first-line approach for the treatment of naïve PLHIV has become the administration of an INSTI with NRTIs [101,141]. While extensive research has not yet been completed to identify the potential deregulation of mitochondria by INSTIs, a recent study assessing metabolic and cellular activity in immune cells from treated PLHIV and HIV-negative controls revealed that ART was able to restore the metabolic profile of B cells, NK cells, and CD8^+^ T cells, but not in CD4^+^ T cells. Furthermore, CD4 T cells showed a more severe shut down in cellular respiration and a shift to glycolysis from OXPHOS. This was more prevalent in cells from individuals treated with the INSTI dolutegravir (DLG) in triple therapy, compared to non-INSTI ART regimens. Additionally, a comparison of the ex vivo proliferation of CD4^+^ T cells demonstrated repressed proliferation in CD4 T cells from individuals on treatment with INSTIs versus NNRTI- or PI-regimens. Further evaluation of the effect of INSTIs was completed by treating CD4 T cells from uninfected individuals. Both DLG and elvitegravir (EVG), but not raltegravir (RAL), showed significantly decreased cellular respiration in a dose-dependent manner but did not affect ΔΨ_m_ or cellular cytotoxicity. Both drugs induced increased mtDNA copy number and ROS levels, implicating INSTIs as a deregulator of mitochondrial function and immune cell metabolism [64]. While this study highlights potential mitochondrial deregulation by INSTIs, further research is still needed to validate the influence of INSTIs on mitochondria.

Holistically, these findings suggest that regardless of the class of ART treatment, PLHIV using these regimens are at a greater risk of developing premature aging associated with mitochondrial dysfunction, many of which are progressive in nature as treatment time is prolonged. This presents a significant limitation with respect to current HIV treatments due to the need for lifelong treatment adherence. Additionally, the current literature lacks clear evidence for the differential roles of drugs and the relationship between drug mechanism of action and mitochondrial failure due to the profound use of combined HAART. This makes it increasingly difficult to assess and identify mitochondrial deregulation from these drugs independent of additional pharmacological influences.

## 3. Discussion

While ART has transformed the HIV/AIDS global epidemic into a well-controlled chronic disease, PLHIV still demonstrate a wide variety of metabolic and immunological failure phenotypes, contributing to increased morbidity and mortality as well as lower patient quality of life and accelerated aging. As shown in Figure 2, both HIV and ART induce a multitude of injuries to mitochondrial function, including deregulation of ETC respiration and ATP synthesis, mtDNA damage, disruption of ΔΨ_m_, and increased oxidative stress. Upon increased oxidative stress, further mtDNA mutations occur, leading to HIV/ART-induced activation of a cycle of continued mtDNA damage, impaired mitochondrial function, increased oxidative stress, and impaired ATP production and cellular homeostasis.

It is thus important to note the mechanisms of mtDNA maintenance and the process of accumulating deleted mtDNA content. Unlike nuclear DNA, mtDNA synthesis occurs in a continual cycle independent of cellular division. HIV and ART regimens are able to disrupt Pol-γ, which works to form the mtDNA replisome along with several critical enzymes, including Twinkle, mitochondrial topoisomerase I, mitochondrial RNA polymerase, RNase H1, and mitochondrial genome maintenance exonuclease 1. Along with these enzymes, mtDNA synthesis also requires equilibrium of deoxyribonucleotide triphosphates (dNTPs) via either de novo or salvage mechanisms, many of which involve mitochondrial transport pathways. Thus, disruption to a step in mtDNA maintenance will lead to the accumulation of damaged mtDNA content. This process has been shown to contribute to the physiological aging process, as many of the critical effector proteins in mtDNA synthesis and repair (including BER) become disrupted or weakened with aging [142,143,144]. In addition to mitochondrial deregulation from mtDNA mutations and damage, telomeres, which serve as a genomic readout of aging, are also sensitive to HIV/ART-induced DNA damage, which could propagate mitochondrial dysfunction via telomere, p53, and PGC (master mitochondrial regulator) interactions in individuals on prolonged ART therapies [7,51,54,83,84]. Thus, p53 activation, telomere erosions, and mitochondrial dysfunction have been identified as a potential “axis of aging” that may illuminate an underlying mechanism by which aging promotes cellular dysfunction and disease progression.

Another critical factor to consider is the influence of heteroplasmy, since human cells have multiple organellar genomes, arising from both mitochondrial and nuclear-encoded genes. It is well understood that a certain level of mitochondrial mutations are always present at low levels but typically only cause physiological changes in the event that the mutations accumulate over time and reach a threshold at which the ratio of mutated mtDNA versus wild-type mtDNA is sufficient (typically >80%) to cause a disruption to the respiratory chain [142,145]. The presence of heteroplasmy likely plays a critical role in the level of mitochondrial dysfunction required to cause physiological problems.

Mitochondrial processes are also affected by virally encoded proteins that serve to deregulate cell metabolism and apoptosis via disruption of the cell apoptotic signals. HIV-proteins may use inhibiting apoptosis as a means to delay premature cell death to facilitate viral persistence. This process is likely mediated by the progression of HIV and the viral lifecycle. For instance, virally encoded proteins are more likely to be antiapoptotic during acute infection to enable persistent infection but may transition to being proapoptotic in the process of establishing chronic infection [14]. This hypothesis may provide insights into the progressive decline across a wide range of cellular contexts in PLHIV on ART [7,67,83].

A clinical feature of HIV infection is the chronic activation of the immune system and inflammation. Several HIV proteins induce activation of the immune system and while ART reduces the level of immune activation, it does not restore it to that observed in uninfected individuals. Furthermore, the role of ART in the differential effects of immune activation and inflammation is still unclear [146,147]. Interestingly, mitochondrial function has been shown to be critically linked to the activation, proliferation, survival, and effector status of T cells. Specifically, mice with a T-cell specific reduction of a mitochondrial complex III subunit showed normal T cell proliferation ability but a significantly reduced level of mitochondrial ROS production, which inhibited the antigen-specific expansion of T cells [148]. These data implicate mitochondrial ROS signaling in the regulation of T cell activation, indicating a potential molecular link between mitochondrial metabolism and immune activation. Furthermore, evidence suggests that HIV uses mitochondria as an additional reservoir to facilitate infection to new target cells [94].

Aging occurs due to the accumulation of cellular injuries, ultimately leading to disrupted cellular homeostasis, increased oxidative stress, and cellular senescence. A significant amount of evidence is present in the literature linking the degenerative aging process to deregulated mitochondrial function [51,65,149]. Specifically, deregulation of mitochondrial respiration, ATP production, and cell apoptosis contribute to progressive loss of cellular homeostasis. Evidence supports the notion of increased incidence of metabolic disorders and mitochondrial failure with increased age, which have been proposed to underlie the loss of vigor in the elderly [149,150,151,152,153]. The model of increased oxidative stress inducing mtDNA mutations is a central mechanism commonly used to explain the aging phenomenon. Furthermore, the expression of critical proteins involved in mitochondrial biogenesis and mtDNA maintenance, including PGC-1α, ERR-α, NRF-1, and TFAM are significantly downregulated in aging cells or tissues [144,154,155,156,157]. Interestingly, age-linked associations have been made across a wide range of chronic viral infections, including but not limited to HIV, HCV, HBV, and CMV, as evidenced by increased telomere erosion and telomeric DNA damage, mitochondrial dysfunction, and cellular senescence [43,57,158,159,160].

Currently, there is conflicting evidence regarding the role of senescence in viral infection, primarily due to some new data implicating senescence in host antiviral defense via inhibiting viral replication, attracting innate immune cells, and activating cytokine signaling. Alternately, several investigations have shown that senescence is involved in the pathophysiology of viral infections via enhancing viral replication and suppressing antiviral type I IFN activation [160]. For example, RSV can cause DNA damage-induced senescence in lung epithelial and mononuclear cells in mice, ultimately leading to airway tissue remodeling and permanent tissue damage [58]. Additionally, influenza and herpes viruses have been shown to have increased viral replication in senescent cells [160,161,162]. Thus, it is likely that the dual role of senescence in virology is virus- and tissue-specific.

Recently, aging, along with mitochondrial dysfunction, has demonstrated a correlation with disease outcome in individuals infected with SARS-CoV-2, the virus responsible for the current COVID-19 worldwide pandemic [163]. One study using an RNA–GPS method predicted that SARS-CoV-2 RNA localized to mitochondria, similar to that observed for HIV, indicating a possible shared mechanism between these viruses in which mitochondria are central to viral infection [164]. Intriguingly, senescence has also been shown to play a role in the severity/death/pathogenesis of COVID-19 as aged individuals have approximately a 20% higher mortality rate than younger individuals when infected with SARS-CoV-2, likely due to the role of senescence in immune cells (immunosenescence) [160,165]. Cellular senescence is also critically influenced by mitochondrial dysfunction, as evidenced by the presence of impaired mitochondria and shortened telomeres (both of which are impacted by HIV infection and ART treatment) [46,166]. This indicates the likelihood that a variety of viral infections use metabolic and mitochondrial deregulation to instigate accelerated telomeric DNA damage, aging, and cellular senescence.

A recent study examined the potential cellular effects of ART as preventative pre-exposure prophylaxis (PrEP) for healthy individuals. PBMCs from individuals treated with PrEP showed reduced cellular respiration and mitochondrial mass. Additionally, monocyte-derived macrophages from PrEP-treated individuals exhibited increased production of ROS and decreased mitochondrial mass, further implicating ART in inducing mitochondrial dysfunction even in the absence of HIV infection [167]. This is of particular importance considering the target populations that are not HIV-infected but have an increased predisposition to HIV, including individuals using PrEP as well as children of infected mothers receiving PrEP as part of prevention of mother to child transmission programs. Interestingly, discontinuation of HAART has been shown to restore mtDNA copy number, implying a direct role of treatment in mitochondrial compromise [129].

More recent studies have highlighted the potential improvement of mitochondrial toxicity associated with newer formulations, including INSTIs, fusion inhibitors, and coreceptor antagonists [108]; however, more extensive studies need to be completed to identify if these drugs and their combined regimens also reduce the likelihood of inducing mitochondrial compromise. Additionally, current research lacks definitive evidence of the level of deregulation due to either ART or HIV alone since most research examines healthy controls, ART-naïve PLHIV, and ART-treated PLHIV, limiting the information obtained regarding ART treatment outside the context of HIV infection. Furthermore, given the overwhelming use of combined therapy, it is difficult to critically assess the differential roles of ART regimens. This review highlights various mechanisms by which both HIV-encoded proteins and ART treatments damage critical mitochondrial functions; however, a multitude of other mechanisms have been studied but are not mentioned here for brevity [14,68,69,85,107].

**Figure 3:**
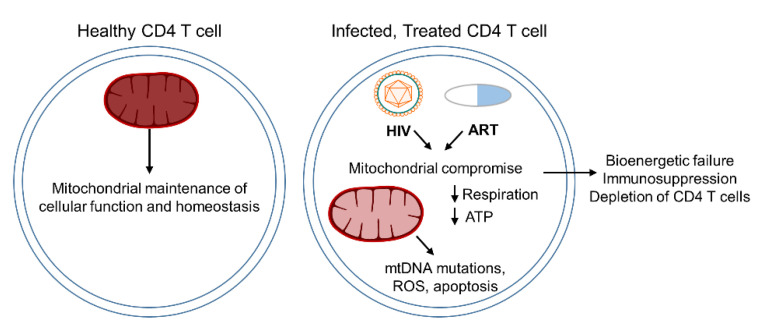
Summary of T cell deficiency in mitochondrial function and premature aging. HIV-infection significantly impairs mitochondrial function with respect to ΔΨ_m_, respiration, ATP synthesis, mtDNA replication, and apoptosis. Each of these factors contributes to immunosuppression, depletion of CD4^+^ T cells, and premature aging in PLHIV.

While advancements concerning ART treatments have provided a wide range of potential therapeutics to PLHIV, it is still critical to examine the mechanisms by which antiretroviral therapies may lead to a progressive decline in cellular functions. This is particularly necessary in the case of PLHIV, who must maintain strict lifelong treatment adherence to prevent viral rebound [109]. Mitochondrial dysfunction induced by both HIV infection and continued ART treatment leads to diverse associated pathologies and potential comorbidities in PLHIV (Figure 3). Having a clearer understanding of the mechanisms by which HIV and ART treatment contribute to mitochondrial dysfunction independently and potentially in a cooperative manner may allow for the improvement of treatment regimens, and a better understanding of disease pathologies associated with immune dysregulated PLHIV.

## Figures and Tables

**Table 1:** Pharmacology of antiretroviral therapy and reported effect on mitochondria [105,109,110].

Drug Class	Mechanism of Action	Mitochondrial Dysfunction	Species and Cell Type Models
NRTIs (Abacavir, Tenofovir)	Prevents viral replication by inhibiting HIV reverse transcriptase	Inhibition of Pol-γ [111]	Human fibroblasts [111,112], PBMCs [106], CD4, and CD8 cells [113], and rat liver cells [30,114]
		Reduction of mtDNA copy number/mitochondrial encoded proteins [106]	
		Reduced lymphocyte proliferation Respiratory chain deficiency Inhibition of ETC complexes [113]	
		ATP reduction [30,114]	
		Increased oxidative stress Decrease in Ψ_m_ [112]	
NNRTIs (Rilpivirine, Efavirenz, Nevirapine)	Prevents viral replication by noncompetitively binding to HIV reverse transcriptase	Respiratory chain deficiency ATP reduction Increased oxidative stress [109]	Human hepatic cells [109], PBMCs [110,115], coronary artery endothelial cells [116], and hepatoma cells [117], and Jurkat T cell line [115]
		Decrease in Ψ_m_ Apoptosis [110,115,116,117]	
PIs (Ritonavir, Darunavir, Atazanavir, Indinavir, Saquinavir)	Prevents viral replication by inhibiting HIV protease	Increased oxidative stress [118]	Human CD4, CD8 [64], macrophage-derived foam cells [119], endothelial [118], hepatoma [117], and hepatic cells [120], and Huh-7.5, 293T, HeLa, and Hepa RG cell lines [121]
		Reduced mtDNA copy number Respiratory chain deficiency Reduced ATP [64,119]	
		Apoptosis [119,122,123]	
INIs (Raltegravir, Dolutegravir, Elvitegravir)	Prevents integration of viral DNA into the host genome by inhibiting HIV integrase enzyme	Respiratory chain deficiency Increased oxidative stress Increased cytoplasmic mtDNA copy number [64]	Human CD4 and CD8 [64]
Fusion Inhibitors (Leronlimab, Ibalizumab, Enfuvirtide)	Prevents viral fusion with target cell membrane by binding to the viral envelope protein gp41	Not identified	N/A
Coreceptor Antagonists (Aplaviroc, Maraviroc, Vicriviroc)	Prevent viral infection by interfering with viral entrance into the cell by blocking the coreceptors, such as CCR5 or CXCR4, on the surface of target immune cells	Not identified	N/A

Note: NRTIs (nucleoside reverse transcriptase inhibitors), human immunodeficiency virus (HIV), mitochondrial DNA (mtDNA), electron transport chain (ETC), mitochondrial membrane potential (Ψ_m_), peripheral blood mononuclear cells (PBMCs), non-nucleoside reverse transcriptase inhibitors (NNRTIs), protease inhibitors (PIs), integrase inhibitors (INIs).
